# Genetic and genomic analysis for cocoon yield traits in silkworm

**DOI:** 10.1038/s41598-020-62507-9

**Published:** 2020-03-30

**Authors:** Shou-Min Fang, Qiu-Zhong Zhou, Quan-You Yu, Ze Zhang

**Affiliations:** 1 School of Life Sciences, Chongqing University, Chongqing, 401331 China; 2 College of Life Science, China West Normal University, Nanchong, 637002 China

**Keywords:** Genomics, Entomology

## Abstract

Domestic species provides a powerful model for examining genetic mechanisms in the evolution of yield traits. The domestic silkworm (*Bombyx mori*) is an important livestock species in sericulture. While the mechanisms controlling cocoon yield are largely unknown. Here, using *B. mori* and its wild relative *B. mandarina* as intercross parents, 100 BC_1_ individuals were sequenced by restriction site-associated DNA sequencing (RAD-Seq). The linkage map contained 9,632 markers was constructed. We performed high-resolution quantitative trait locus (QTL) mapping for four cocoon yield traits. A total of 11 QTLs were identified, including one yield-enhancing QTL from wild silkworm. By integrating population genomics and transcriptomic analysis with QTLs, some favourable genes were revealed, including 14 domestication-related genes and 71 differentially expressed genes (DEGs) in the fifth-instar larval silk gland transcriptome between *B. mori* and *B. mandarina*. The relationships between the expression of two important candidate genes (*KWMTBOMO04917* and *KWMTBOMO12906*) and cocoon yield were supported by quantitative real-time PCR (qPCR). Our results provide some new insights into the molecular mechanisms of complex yield traits in silkworm. The combined method might be an efficient approach for identifying putative causal genes in domestic livestock and wild relatives.

## Introduction

Domestication played a central role in formulating Charles Darwin’s theory of evolution^1^. Since domestication, crops and animals have been under artificial selection, largely directed towards increasing yield, which provides useful model systems to characterize the genetic mechanisms of yield traits. In recent years, domestication-related genes for yield traits have been cloned from several crops^2,3^. The domestic silkworm (*Bombyx mori*) is an economically important insect. Compared with its wild relatives, long-term artificial breeding and selection have resulted in a high cocoon yield of domestic silkworm^4^. However, the molecular mechanisms associated with silk yield remain largely undefined in silkworm^5^.

QTL mapping is a method for locating the genetic loci within a whole genome that might contain genes of small effect contributing to yield traits. In domestic silkworm, QTL mapping has been conducted using traditional molecular markers, such as amplified fragment length polymorphisms (AFLPs) and simple sequence repeats (SSRs)^6–11^. However, due to the limitation of marker numbers, it is relatively difficult to narrow QTL regions of interest^12^. With the advance of next-generation sequencing (NGS) technology, restriction-site-associated DNA sequencing (RAD-Seq) has been developed, which scans short sequence regions surrounding all restriction sites for a given restriction endonuclease^13^. A large number of single nucleotide polymorphism (SNP) markers are available that can be used for constructing a high-density genetic map, QTL mapping and population genomic analyses^14–17^. In *Plutella xylostella*, 2,878 segregating RAD alleles helped construct a linkage map and match linkage groups with homologous chromosomes of *Bombyx mori*^14^. In rainbow trout, a three-generation F_2_ mapping family was genotyped using RAD-Seq to identify 4874 informative SNPs^18^. Thus, RAD-Seq is a powerful tool for high-density genetic map construction and QTL mapping^13^.

A clear limitation of QTL mapping is that the confidence intervals are usually large, potentially harbouring up to dozens of genes. Pinpointing candidate gene by means of QTL analysis alone is often extremely challenging^19^. In crops, one way to finely dissect QTL regions is to breed advanced intercross lines or nearly isogenic lines (NILs)^3,19–21^. However, it is difficult to maintain large mapping populations over multiple generations, and this incurs great cost in terms of time in livestock. Searching for selective signals in the whole genome of domestic species becomes more convenient^4,19^. Thus, the combination of population genomics and QTL mapping should be an efficient approach for identifying causal genes of domestication-related yield traits^22^.

Increasing yield is one of principal purposes of animal breeding programs. Furthermore, genetic diversity is a fundamental criterion of high-yield breeding. Generally, the genetic improvement of *B. mori* has mainly been restricted by the domestic populations^23,24^. *B. mori* has been domesticated from the wild mulberry silkworm *B. mandarina* for at least 5000 years^4,25^. Utilization of wild silkworm might provide a more powerful way to exploit novel genetic resources^23,26–28^. In this study, we used the domestic silkworm Xiafang (D_XF) and the wild silkworm (W_AK) as intercross parents and employed their backcross progenies (BC_1_M) as the mapping population. Using RAD-Seq technology, the yield-related loci were mapped in the whole genome of the domestic and wild silkworms. Combining with population genomics and developmental transcriptomic data of the silk gland, integrated analyses were applied to reveal those yield-related loci. It provides a good opportunity for exploring yield-related genes and molecular mechanisms in domestic and wild silkworms, which might be used for high-yield breeding with genome editing, genetic knockdown and overexpression technologies^29,30^.

## Results

### Phenotypic variation in the mapping population and correlation between yield traits

In this study, the domestic silkworm strain Xiafang (D_XF), which is widely used in sericulture production in China, and the wild silkworm (W_AK) were used as the parental mapping lines. All four cocoon yield-related traits showed significant differences between D_XF and W_AK (Table 1). In particular, the cocoon shell weight (CSW) and whole cocoon weight (WSW) of the domestic strain Xiafang reached to 4.68 and 2.76 times those of W_AK, respectively. The phenotypic values of the traits measured in the BC_1_ population were nearer to those of the recurrent parent D_XF (Table 1).

**Table 1:** Phenotypic values of parental lines and the backcross population (BC_1_) for cocoon yield traits.

Trait	D_XF	W_AK	BC_1_ population		
	(Mean ± Std)	(Mean ± Std)	Mean ± Std	Min^a^	Max^b^
WCW	1.678 ± 0.188	0.639 ± 0.202	1.367 ± 0.228	0.967	1.904
CSW	0.379 ± 0.024	0.079 ± 0.019	0.300 ± 0.041	0.192	0.398
CSR	22.768 ± 2.276	12.906 ± 2.185	22.258 ± 3.104	10.075	29.662
PW	1.299 ± 0.179	0.559 ± 0.185	1.067 ± 0.209	0.728	1.712

D_XF, the domestic silkworm strain Xiafang. W_AK, wild silkworms collected from Ankang City, China. The mean value of yield traits was obtained from 20 male and 20 female individuals. Significant differences of the four traits between D_XF and W_AK were observed (ANOVA, P <0.001). Std, standard deviation. aMin and bMax represent minimum and maximum values. WCW, whole cocoon weight; CSW, cocoon shell weight (g); CSR, cocoon shell ratio (%); PW, pupal weight (g).

The correlation between the four cocoon yield traits was evaluated (Table 2). Significantly positive correlations were observed among WCW, CSW, and pupal weight (PW). The highest correlation was shown between WCW and PW (r = 0.99, *P* < 0.01). The cocoon shell ratio (CSR) was positively correlated with CSW (r = 0.34, *P* < 0.01) but negatively correlated with WCW (r = −0.61, *P* < 0.01) and PW (r = −0.73, *P* < 0.01). In addition, we compared the correlations between cocoon yield traits and sex. The results indicated that WCW and PW were positively correlated with female, i.e., female individuals exhibited higher WCW and PW values than males. The cocoon shell ratio was negatively correlated with female (r = −0.73, *P* < 0.01). No significant correlation was observed between cocoon shell weight and female/male was observed.

**Table 2:** Correlation coefficients among the four cocoon yield traits and sex in the backcross population.

Trait	WCW	CSW	CSR	PW
CSW	0.53^**^			
CSR	−0.61^**^	0.34^**^		
PW	0.99^**^	0.37^**^	−0.73^**^	
Sex	0.80^**^	0.18	−0.73^**^	0.83^**^

Average phenotypic values from 100 BC1 individuals were used for the Pearson correlation test. **P <0.01.

### Genetic linkage map and QTL mapping

**Figure 1:**
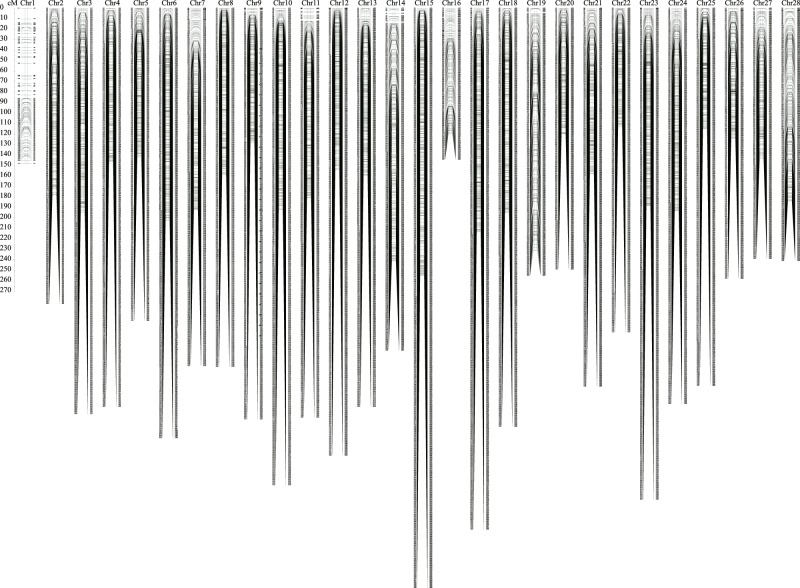
High-density SNP linkage map of the silkworm.

A total of 100 BC_1_ individuals were sequenced using RAD-Seq technology (Supplementary Table S1). The number of RAD tags for the 100 BC_1_ individuals ranged from 720,477 to 4,622,071 with an average of 2,230,620 (Supplementary Table S2). After quality filtration, a total of 49.69 million high-quality SNPs were detected (Supplementary Table S3). The whole genomes of the intercross parents have been resequenced with NGS technology^31^. The average sequencing depth was 14.42-fold in the female parent (D_XF) and 12.83-fold in the male parent (W_AK). In the mapping parents, 5,748,787 homozygous and polymorphic SNP markers were detected. Through filtration and genotyping in the BC_1_ mapping population, 51,418 effective markers were identified and used for further linkage map construction. A total of 28 genetic linkage groups were constructed with MSTmap software (Fig. 1 and Supplementary Table S4) and found to exhibit one-to-one correspondence with the silkworm chromosomes^32^. The linkage map contained 9,632 markers and covered 4,764.96 cM, with a range of 112.31 cM to 254.83 cM.

**Figure 2:**
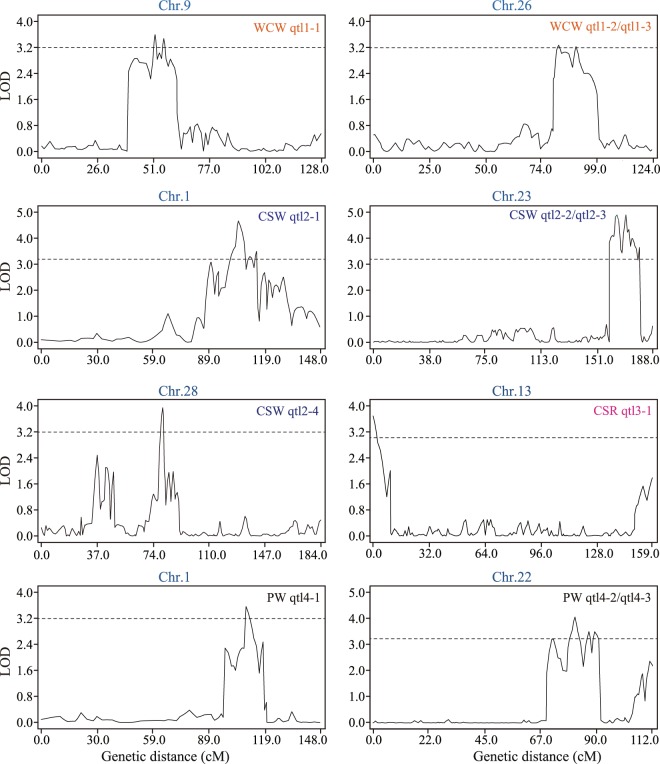
QTL distribution on a continuous chromosome determined from composite interval mapping with the BC_1_ population.

A total of 11 cocoon yield-related QTLs were identified on 7 chromosomes using the composite interval mapping (CIM) algorithm (Fig. 2 and Table 3). The contribution rate (R^2^) of a single QTL varied from 9.52% to 13.68%, and qtl2-2 had the largest effect. Most of the QTLs showed positive additive effects, implying a higher value for the trait conferred by the allele of the domestic strain Xiafang (Table 3), while qtl2-4 (CSW) showed negative additive effects. Interestingly, the 95% confidence intervals (95% CI) of qtl2-1 (CSW) and qtl4-1 (PW) were 99.90–107.52 cM and 108.52–110.59 cM on chromosome 1, respectively (Table 3). It was suggested that CSW and PW traits showed a certain genetic relatedness.

**Table 3:** QTLs detected through composite interval mapping in the BC_1_ population.

Trait	QTL name	Chr	LOD peak	LOD peak position(cM)	Left marker	Right marker	95% CI(cM)	Additive effect	R^2^(%)
WCW	qtl1-1	9	3.60	51.81	mk14610	mk14818	51.54–56.30	0.16	10.67
WCW	qtl1-2	26	3.27	82.01	mk49168	mk49084	81.00–86.29	0.15	9.60
WCW	qtl1-3	26	3.23	89.71	mk49031	mk49007	89.15–90.68	0.15	9.52
CSW	qtl2-1	1	4.66	104.51	mk38	mk76	99.90–107.52	0.03	13.48
CSW	qtl2-2	23	4.89	164.21	mk41983	mk41914	159.04–167.58	0.03	13.68
CSW	qtl2-3	23	4.89	170.21	mk41872	mk41877	169.09–173.23	0.03	13.07
CSW	qtl2-4	28	3.94	79.91	mk50998	mk51009	78.76–80.40	−0.10	10.27
CSR	qtl3-1	13	3.69	0.01	mk26265	mk26369	0–1.86	0.04	12.51
PW	qtl4-1	1	3.54	108.61	mk76	mk75	108.52–110.59	0.15	12.70
PW	qtl4-2	22	4.07	81.21	mk40213	mk40245	78.98–83.49	0.30	12.54
PW	qtl4-3	22	3.50	86.81	mk40227	mk40330	85.72–90.72	0.30	10.96

Chr, chromosome; 95% CI, 95% confidence interval; R^2^, percentage of phenotypic variation explained by the QTL.

### Identification of putative candidate genes associated with QTLs

**Figure 3:**
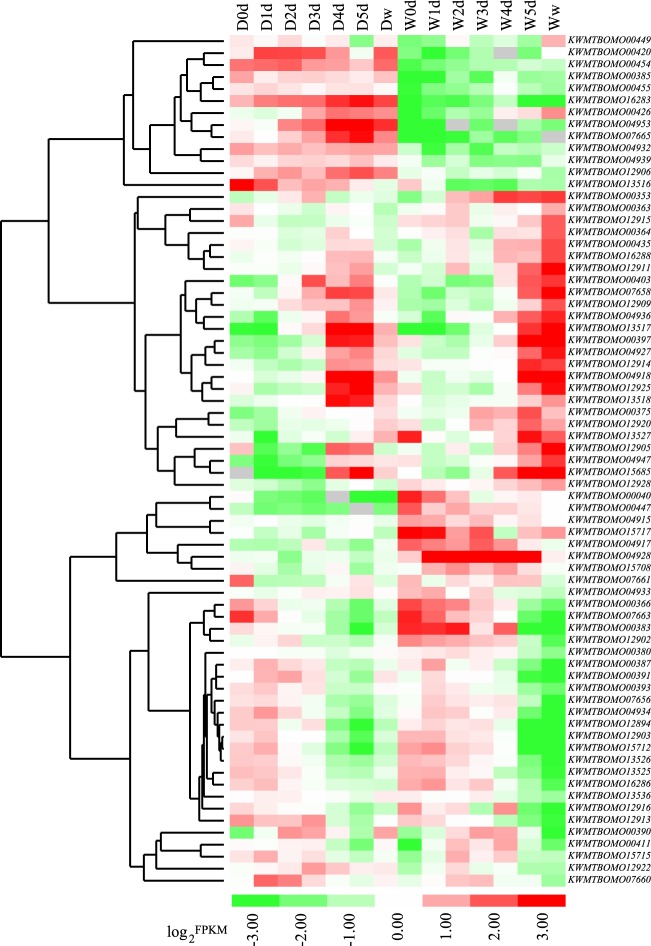
Hierarchical clustering of the differentially expressed genes in the QTL regions. In the sample names, D and W indicate domestic and wild silkworms, respectively. 0d: beginning of the fifth-instar larvae; 1d to 5d: day 1, 2, 3, 4, and 5 of the fifth-instar larvae, respectively; w: beginning of the wandering stage.

Based on the transcriptome of the fifth-instar larval silk gland and the new genome assembly of silkworm^33^, 194 protein-encoding genes with expression signal (FPKM > 1) were identified in the 11 QTLs (Supplementary Table S5). The number of genes ranged from 8 (qtl1-3 and qtl2-4) to 59 (qtl2-1). Among the expressed genes, binding, organic cyclic compound binding and heterocyclic compound binding were the most enriched GO terms (Supplementary Fig. S1a). Pathway enrichment analysis indicated that metabolic pathway, peroxisome and longevity regulating pathway contained more genes (Supplementary Fig. S1b). To pinpoint candidate genes, the developmental transcriptome of the fifth-instar larval silk gland were compared between the domestic and wild silkworms, including seven stages from the initiation of the fifth instar to wandering (Supplementary Table S5). In total, 71 differentially expressed genes (DEGs) were identified during the seven developmental stages (Fig. 3 and Supplementary Table S5).

**Figure 4:**
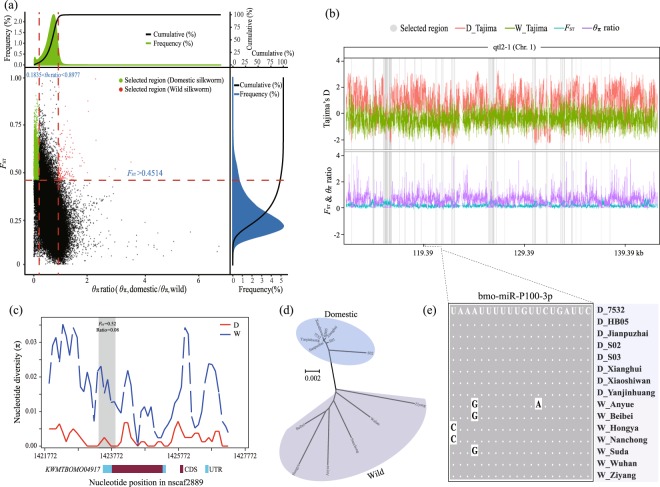
Genomic regions and candidate genes showing signals of a selective sweep in QTLs. (**a**) Distribution of ratio (*θ*_π, domestic_/*θ*_π, wild_) and *F*_ST_ of 500-bp windows with 500-bp steps in the whole genome. At a 5% significance level (corresponding to *θ*_π_ ratio = 0.1835 and *F*_ST_ = 0.4514), the selected windows were considered as candidate regions with signals of a selective sweep for domestic silkworm (green dots). Red dots represent selected regions in the wild populations. (**b**) Example of qtl2-1 on chromosome 1 with signals of a selective sweep (grey regions) in domestic silkworms. (**c**) Example of a gene with a signal of a selective sweep (grey region) in qtl1-1. Gene structure is shown at the bottom. CDS: coding sequence; UTR: untranslated regions. D: domestic silkworm; W: wild silkworm. (**d**) Neighbour-joining (NJ) tree of *KWMTBOMO04917*. The NJ tree of the DNA sequences used in (**c**) was reconstructed with MEGA6.0^37^ using a Kimura 2-parameter model, 500 replicates and pairwise deletion. (**e**) The miRNA showed sequence diversification between the domestic and wild populations.

A combination of selective sweeps and QTL mapping analyses might be an efficient approach for pinpointing the candidate genes associated with domestication^34,35^. In the silkworm, cocoon yield traits are also under control of domestication. Whole-genome resequencing of eight silkworm strains and seven wild silkworms from ecologically different regions has been conducted in our laboratory^36^. The selective sweep regions were identified in the whole genome, which showed extremely low heterozygosity and high *F*_ST_ values relative to wild populations (Fig. 4a). As shown in Fig. 4b, selective sweep analysis revealed the genetic complexity underlying some QTLs. The qtl1-1 on chromosome 9 and qtl2-1 on chromosome 1 co-localized with 7 and 6 selective sweep regions, respectively (Supplementary Table S6). Conversely, four QTLs (qtl2-3, qtl3-1, qtl4-1, and qtl4-3) had a simpler genetic profile and contained only one sweep region with one gene, respectively. In total, 14 domestication-related genes were characterized within the QTL regions (Supplementary Table S6), which might provide multiple valuable candidates for understanding molecular mechanisms of cocoon yield.

**Figure 5:**
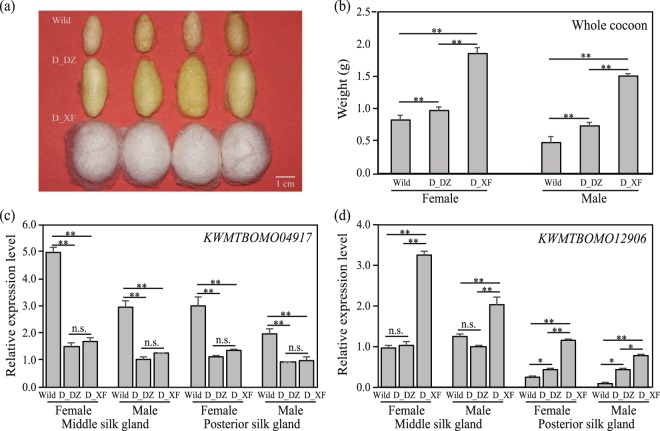
qPCR validation of candidate genes in domestic and wild silkworms. (**a**) Comparison of cocoon of the domestic strain Dazao (D_DZ), Xiafang (D_XF) and wild silkworm. (**b**) Whole cocoon weight of the wild silkworm and domestic strains used for qPCR analysis. (**c**,**d**) qPCR validation of the interesting candidate genes in the silk gland on day 2 of the wild and domestic silkworms. The design of the specific primers and the qPCR strategy were similar to our previous study^38^. The primer sequences are listed in Supplementary Table S7. One-way analysis of variance (ANOVA) was used to determine significant differences between any two samples. *P < 0.05; **P < 0.01; n.s.: not significant.

*KWMTBOMO04917*, encoding 5-aminolevulinate synthase (ALAS; E.C.2.3.1.37), was one of the putative genes under artificial selection (Fig. 4c,d) and was differentially expressed between the domestic and wild silkworms at five out of the seven developmental stages of the fifth-instar silk gland (Supplementary Table S5). To understand the relationship of *KWMTBOMO04917* with silk yield, its transcriptional level was detected by quantitative real-time PCR (qPCR) in the silk gland of high-yield Xiafang, low-yield Dazao (D_DZ) and the wild silkworm (Fig. 5a,b). Both qPCR and RNA sequencing indicated that the expression of *KWMTBOMO04917* was higher in the wild silkworm than in the domestic silkworms (Fig. 5c and Supplementary Table S5). *KWMTBOMO12906*, encoding for DNA polymerase epsilon (Pol ε) subunit 4, was one of the few DEGs that was highly expressed in the domestic silkworms and showed differential expression at four out of the seven developmental stages (Fig. 3 and Supplementary Table S5). The qPCR result indicated that the expression of *KWMTBOMO12906* in the posterior silk gland (PSG) was positively correlated with cocoon yield (Fig. 5d and Supplementary Fig. S2). These results suggested that the genes identified via integrated methods might be important candidates controlling cocoon yield.

### Yield-enhancing QTLs from wild silkworm

Based on QTL mapping, it was suggested that qtl2-4 from the wild parent was related to an increase in the trait score (Table 3). The qtl2-4, controlling cocoon shell weight, was located on chromosome 28 (Fig. 2 and Table 3). This QTL contained 8 protein-coding genes showed expression signals (FPKM > 1) in the silk gland of fifth-instar larvae (Supplementary Table S5). *KWMTBOMO16286*, *KWMTBOMO16288* and *KWMTBOMO16283* showed differential expression between the domestic and wild silkworms. Especially, *KWMTBOMO16283* presented higher expression in silk gland of the domestic silkworm than wild silkworm during all the fifth-instar stages.

### MicroRNAs in QTL regions

In the domestic silkworm, miRNAs were characterized in the fifth-instar posterior silk gland using NGS technology^39^. Based on the previous annotation, 39 miRNAs were found in the QTL regions (Supplementary Table S8). To understand their potential roles, the interaction of the miRNAs and the 3′ untranslated region (3′ UTR) of silk fibroin genes was predicted with miRanda software (Supplementary Table S8). The results indicated that *Fib-L* (fibroin light chain) is a putative target of bmo-miR-P009-3p, bmo-miR-P1416-3p and bmo-miR-P581-5p. More interestingly, bmo-miR-P100-3p presented some SNPs between the domestic and wild populations (Fig. 4e). In addition, *KWMTBOMO04917* is one of the putative targets of bmo-miR-P100-3p in domestic silkworms. However, due to the SNP (A to G) in W_Anyue, W_Beibei and W_Suda, the orthologous bmo-miR-P100-3p exhibited no target site in the 3′ UTR of *KWMTBOMO04917* in those wild silkworms.

## Discussion

In this study, the domestic silkworm and its wild relative *B. mandarina* were used for mapping parents. A linkage map of 9,632 SNP markers was constructed, and 11 QTLs related to WCW, CSW, CSR, and PW traits were identified (Figs. 1 and 2). In the previous study, an integrated consensus map contained 692 unique SSR sites was developed^7^. Based on the linkage map, at least six QTLs involved in WCW, CSW, CSR, and PW were detected, which located in chromosomes 1, 18, 19, 21, 22, and 23. In addition, using the pooling sequencing-based methodology, 8 SNPs linked with CSW trait were also identified, which located on chromosomes 11, 22 and 23^12^. These results suggested that the silk yield traits were mainly located on some specific chromosomes. However, different studies might find diverse QTLs on the same chromosome. For instance, SNP marker193648 on chromosome 23^12^ was not included in qtl2-2/ qtl2-3 regions related to CSW in present study. It was indicated that silk yield is controled by multiple QTLs, and different mapping parents might obtain some novel loci.

Silk gland is responsible for silk protein synthesis and excretion in silkworm^40^. In this study, the developmental transcriptome of the fifth-instar silk glands was compared between the domestic and wild silkworms, in which *KWMTBOMO04917* and *KWMTBOMO12906* were DEGs in multiple stages (Supplementary Table S5). At the same time, *KWMTBOMO04917* was one of the domestication-related genes that showed higher expression in the middle and posterior silk gland of the wild silkworm compared to those of the domestic strains (Fig. 5c). However, no significant difference was found in domestic strains Xiafang and Dazao, which might be affected by the direction of artificial selection, which has resulted in interspecies differences in gene expression and low within-species variance^41^. *KWMTBOMO04917* encodes 5-aminolevulinate synthase (*BmALAS*), which may function as a homodimer and converts glycine into 5-aminolevulinate in a single step^42^. A previous study revealed that glycine was one of the major amino acid residues in the silk fibroin heavy chain^43^. Due to the lower expression of *BmALAS*, more glycines might be used for the synthesis of silk proteins in domestic silkworm. In addition, the expression of *KWMTBOMO12906*, encoding DNA polymerase epsilon (Pol ε) subunit 4 (POLE4), was positively related to cocoon yield (Fig. 5d and Supplementary Fig. S2). The protein sequences of POLE4 were identical among Dazao, D_XF, and W_AK (Supplementary Fig. S3). Previous studies suggested that the synthesis of silk proteins is proportional to the DNA dosage per unit silk gland cell^44,45^. We assumed that the higher expression of POLE4 in the high-yield strain might increase the DNA content in silk gland cells and result in an increased cocoon yield.

Except for *B. mori*, wild silkworm might also possess genes potentially involved in silk improvement. In this study, one yield-enhancing QTLs (qtl2-4) was identified from wild silkworm (Table 3), which provides an opportunity to mine favourable genes from wild relatives. Only 8 protein-encoding genes were identified in qtl2-4, in which 3 genes showed differential expression in developmental transcriptomes of the fifth-instar silk glands between the domestic and wild silkworms (Supplementary Table S5). *KWMTBOMO16283* has higher expression in the domestic silkworm than wild silkworm during all the fifth-instar stages. While its function is unknown through homologous search in GenBank (Supplementary Table S5). It is worth exploring whether *KWMTBOMO16283* plays roles in mediating the biosynthesis and secretion of silk proteins.

MicroRNAs (miRNAs) recognized as important regulators of gene expression in higher eukaryotes^46^. In recent years, several miRNAs have been reported to control rice grain yield^47,48^. In the domestic silkworm, miRNAs were characterized in silk gland^39^. Based on the annotation, 39 miRNAs were found in the QTL regions (Supplementary Table S8), which may potentially regulate the genes related to protein synthesis and processing^39^. It was found that no miRNA within QTLs was located in the candidate selective sweep regions (Supplementary Table S6). While, three SNPs was detected in bmo-miR-P100-3p between the domestic and wild populations (Fig. 4e). Especially, the SNP (G to A) can affect formation and stability of miRNA-target duplex, which may down-regulate the expression of *KWMTBOMO04917* in the domestic silkworm (Fig. 5c). In addition, some differentially expressed miRNAs were found in the silk gland among three silkworm strains with differential silk production^49^. It was suggested that differential expression of miRNAs may also affect transcriptional and translational level of the target genes. Further studies are needed to determine whether those miRNAs within the QTLs would influence expression of the target genes and cocoon yield traits in silkworm.

## Conclusions

In this study, 11 QTLs related to cocoon yield traits were mapped to 7 chromosomes in silkworm. Some of those traits shared the adjacent genomic regions, which shows a certain genetic relatedness. Integrated QTL mapping, population genomics, and transcriptional profiling analyses allowed us to implicate some potential causative genes for cocoon yield traits. Simultaneously, one trait-improving QTL allele (qtl2-4) from the wild parent was identified, which will help to explore yield-enhancing genes in wild silkworms. The top candidate genes will be particularly interesting targets for experimental verification.

## Materials and Methods

### Silkworm rearing and sample preparation

The domestic silkworm was reared on fresh mulberry leaves at 25 ± 1 °C and 75% ± 3% relative humidity (14 hours light: 10 hours dark). The wild silkworm was collected from Ankang City, Shaanxi Provence in China. An F_1_ intercross was performed from a female of the domestic silkworm Xiafang (D_XF) and a male of the Ankang wild silkworm (W_AK). We generated backcross (BC_1_M) progeny through single-pair matings between an F_1_ male with a Xiafang female. Unhealthy individuals were discarded during rearing. On the eighth day after wandering, the cocoon yield traits of each individual were investigated, including whole cocoon weight (WCW), cocoon shell weight (CSW), cocoon shell ratio (CSR), and pupal weight (PW). Thereafter, the pupae were stored at −80 °C for genomic DNA isolation.

### RAD sequencing

Genomic DNA was extracted from 100 BC_1_ individuals using the classical phenol/chloroform method^50^. The quality of DNA was checked with a NanoDrop 2000 spectrophotometer (Thermo Scientific, USA), and through 1% agarose gel electrophoresis. DNA from each individual was digested with the restriction endonuclease *Eco*R I. Solexa P1 adapters were added to the digested fragments. The ligated DNA samples were pooled and sheared using a Covaris S-Series ultrasonicator (Covaris) to an average size of 500 bp. The sheared samples were separated in a 1.5% agarose gel, and DNA fragments of 300 to 700 bp were isolated and enzymatically blunted. The Klenow fragment (exo^-^; New England Biolabs) was then used to add an adenine to the 3′ end. An adapter with divergent ends (P2 adapter) was ligated to conduct selective PCR. The libraries were run for sequencing of paired-end reads (100 bp) on the Illumina HiSeq. 2000 Genome Analyzer platform (Novogene, Beijing, China).

### Reads mapping to the reference genome

The genomes of the mapping parents D_XF and W_AK were resequenced in a previous study by our group and deposited in the ENA database (The European Bioinformatics Institute) under accession number PRJEB5458^31^. The raw reads of the mapping population and parents were filtered by removing adaptor sequences and low-quality sequences containing >10% poly-N or >50% bases with Phred quality scores ≤5. The genome version 3.0 of the silkworm was downloaded from SilkBase (http://silkbase.ab.a.u-tokyo.ac.jp). The clean reads were mapped to the reference genome using Burrows-Wheeler Alignment (BWA) tool^51^, with the following parameters: aln -o 1 -m 100000 -t 4 -l 32 -i 15 -q 10. Reads that were not uniquely mapped were discarded and not considered in further analysis.

### SNP detection and genetic marker development

Alignments were piped with SAMtools v0.1.18^52^ and reformatted into BAM and pileup files for SNP identification, with the following parameters: -m 2 -F 0.002 -d 1000. Among the homozygous SNPs in the intercross parents, polymorphic SNP markers were developed. To be considered for genotyping design, an SNP had to exhibit minimum sequencing coverage of 4× in the intercross parents and 2× in BC_1_ individuals. SNPs that were consistently identified in parents and the progenies were retained. Some abnormal bases existed in offspring rather than parents, which were treated as a deficiency in the offspring. To maintain the integrity of markers, the degree of genotype coverage in offspring was over 85%. If the distribution of markers in offspring showed segregation distortion (chi-square test, *P* < 0.001)^53^, it was discarded. After filtration based on abnormal bases, integrity and segregation distortion, the high-quality markers were used for genotyping.

### Linkage group construction and QTL analysis

The genetic linkage map was constructed using MSTmap software^54^ and visualized with Map Draw V2.1^55^. The composite interval mapping (CIM) algorithm implemented in WinQTLCart v2.5 software (http://statgen.ncsu.edu/qtlcart/WQTLCart.htm) was employed to scan QTLs. The CIM analysis was based on Model 6, with a 1 cM step size, forward and backward stepwise regression, and five control markers. The LOD threshold was determined based on 1,000 permutations with a genome-wide level of significance of 5%. Sex as a categorical trait was used as a factor that can be “regressed out” in regression analysis.

### Screening the genes under artificial selection within QTL regions

The putative candidate genes were explored in the genomic region located within the boundary markers of QTLs and its 200 kb flanking regions. Gene Ontology (GO) enrichment analysis was performed with BLAST2GO^56^. Pathway analysis of the candidate genes was performed with the KOBAS 2.0 online tool^57^. Whole-genome resequencing data of eight domestic and seven wild individuals could be archived from National Genomics Data Center (NGDC, http://bigd.big.ac.cn/) under accession number PRJCA002125. These deeply resequenced genomes (approximately 40-fold coverage) were aligned to the silkworm reference genome using BWA-MEM v0.7.7 with the default parameters^58^. We conducted a series of procedures before SNP calling, including removing PCR duplicates with Picards v1.41, realignment of indels using GATK v3.4^59^. SNPs and insertion/deletion polymorphisms (INDELs) were identified with the GATK, SAMtools, and FreeBayes tools^52,60,61^. The shared variants supported by at least five reads were used in the subsquent analysis of population genetics. To identify the genes that may have been subjected to selection during domestication, we calculated the ratio of genetic diversity (*θ*_π, domestic_/*θ*_π, wild_) and the population differentiation statistic (*F*_ST_)^62,63^. We used an empirical procedure and selected windows with a 5% significance level of the *θ*_π_ ratio and a 5% significance level of *F*_ST_ as candidate regions with strong signals of a selective sweep for the domestic silkworm^17,64^. In the QTL regions, genes showing evidence of a selective sweep were considered as important candidates^35,64^.

### Developmental transcriptome analysis of the fifth-instar silk gland

The domestic strain Xiafang and the wild silkworm from Chongqing in China were used for comparative transcriptome analysis. The rearing method of the wild silkworm was similar to that applied in our previous study^38^. Seven time points of fifth-instar larvae were selected in the wild silkworm, including beginning (0d), day 1 (1d), day 2 (2d), day 3 (3d), day 4 (4d), and day 5 (5d) of the fifth-instar and beginning of wandering (w). Due to the longer feeding period of the domestic silkworm, the corresponding time points were averagely partitioned in relation to those of the wild silkworm. The intact silk glands of three individuals were dissected at each time point. Each sample was repeated two times for RNA sequencing. Transcriptome sequencing and analysis were performed similarly to our previous study^38^. The analysis of differential expression was conducted at the same time point between the domestic and wild silkworms using the edgeR package^65^. The *P*-value was adjusted by the method of Benjamini & Hochberg^66^. Differentially expressed genes (DEGs) were identified according to threshold values of an adjusted *P*-value <0.05 and a|log_2_ fold-change|>1.

### Prediction of microRNA-target duplexes

Based on miRNA annotation in silkworm^39^, miRNAs located in QTL regions were identified. The putative targets of the miRNAs were mainly focused on silk fibroin heavy chain (*Fib-H*), light chain (*Fib-L*) and *P25*, and the QTL-harbored DEGs in developmental transcriptomes of the fifth-instar silk gland between the domestic and wild silkworms. The 3′ untranslated regions (3′-UTRs) of *Fib-H*, *Fib-L* and *P25* were retrieved from BB988047.1, NM_001044023.1, and X04226.1, respectively, in GenBank. The target site of 3′-UTR region was predicted with miRanda v3.3a software^67^ based on the following parameters: score cutoff = 140, energy cutoff ≤ −7.0, gap opening: −9.0, and gap extension −4.0.

### Ethics approval and consent to participate

Experiments were conducted in accordance with the protocol approved by the Institutional Animal Care and Use Committee of the Chongqing University (permit number CBE-A201405010).

## Supplementary information

Supplementary figures and tables

Supplementary Table S5

## Data Availability

RAD sequencing datasets of 100 BC_1_ individuals were available at NCBI Sequence Read Archive (SRA) database under accession number PRJNA453838. Whole-genome resequencing data and the developmental transcriptome of the fifth-instar larval silk gland could be retrived from NGDC (http://bigd.big.ac.cn/) under the accession numbers PRJCA002125 and PRJCA001835, respectively.
