# Supplementary material for: Genetic and genomic analysis for cocoon yield traits in silkworm
Source: Sci Rep. 2020 Mar 30;10:5682. doi: 10.1038/s41598-020-62507-9 (PMC7105477; doi:10.1038/s41598-020-62507-9)
Supplement: Supplementary file 1 — Supplementary figures and tables [file 41598_2020_62507_MOESM1_ESM.pdf]

Supplementary information

Genetic and genomic analysis for cocoon yield traits in silkworm

Shou-Min Fang[1,2], Qiu-Zhong Zhou[1], Quan-You Yu[1,*] , Ze Zhang[1]

[1]School of Life Sciences, Chongqing University, Chongqing 401331, China.

[2]College of Life Science, China West Normal University, Nanchong 637002, China.

*Corresponding authors: yuqy@cqu.edu.cn

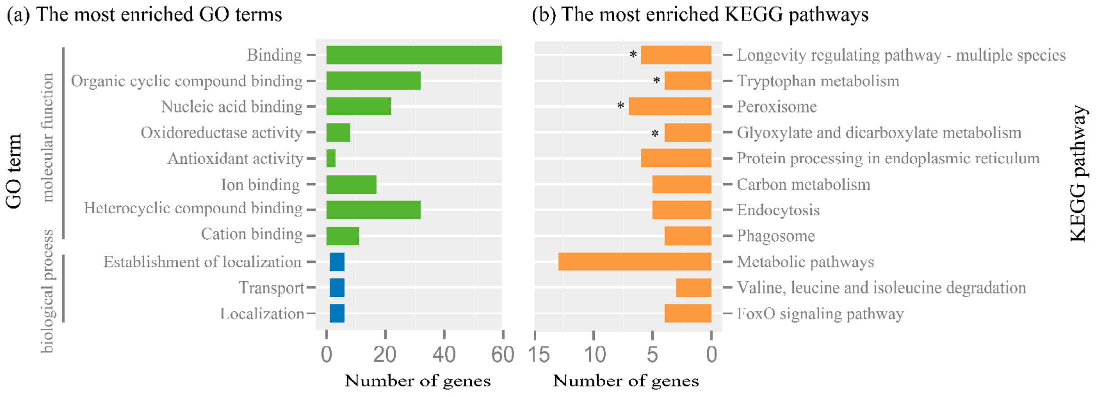

Supplementary Fig. S1 The most enriched Gene Ontology and KEGG categories of the expressed genes within QTL regions. (a) The most enriched GO terms. (b) The most enriched KEGG pathways. **P* <0.05, significantly enriched categories.

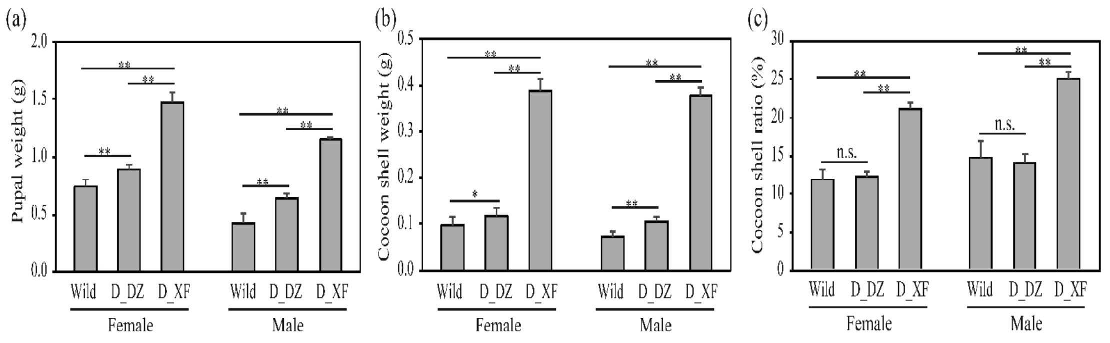

Supplementary Fig. S2 Comparison of cocoon yield traits in the domestic and wild silkworms used for qPCR validation. (a) Comparison of pupal weight. (b) Cocoon shell weight. (c) Cocoon

shell ratio. One-way analysis of variance (ANOVA) was used to determine significant differences between any two samples. $^{*}P < 0.05$; $^{**}P < 0.01$; n.s.: not significant.

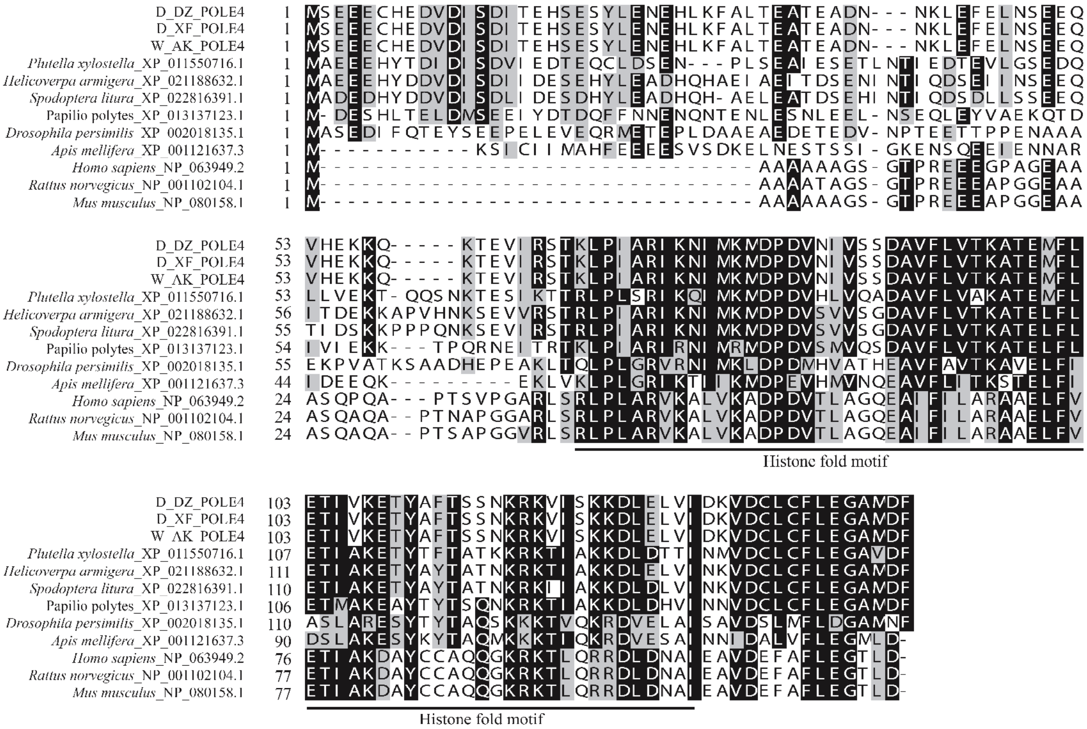

Supplementary Fig. S3 Alignment of the amino acid sequences of DNA polymerase epsilon subunit 4 (POLE4). POLE4 sequences of the other species were retrieved from GenBank (https://www.ncbi.nlm.nih.gov/). Residues in black boxes indicate sequence identity. Residues in grey boxes indicate conservative substitutions. The conserved histone fold motif is underlined.

Supplementary Table S1 Sequencing information for each individual used for QTL mapping.

Samples	Raw reads	Raw Base (bp)	Clean Base (bp)	Error Rate (%)	Q20 (%)	Q30 (%)	GC content (%)
BC1-01	3,029,964	605,992,800	550,050,800	0.03, 0.03	98.47, 96.91	94.37, 91.98	38.83, 39.02
BC1-02	3,715,760	743,152,000	707,035,000	0.03, 0.03	98.05, 96.06	93.49, 90.85	35.98, 36.20
BC1-03	4,566,401	913,280,200	874,589,000	0.03, 0.03	97.64, 96.30	92.28, 90.72	35.27, 35.72
BC1-04	4,565,707	913,141,400	870,893,400	0.03, 0.03	97.61, 96.23	92.26, 90.63	35.28, 35.71
BC1-05	3,116,172	623,234,400	596,583,400	0.03, 0.03	98.03, 96.01	93.42, 90.72	35.94, 36.11
BC1-06	2,284,044	456,808,800	431,993,200	0.03, 0.03	97.72, 96.50	92.54, 91.17	35.35, 35.72
BC1-07	2,943,778	588,755,600	558,651,600	0.03, 0.03	98.08, 96.10	93.57, 90.95	35.89, 36.06
BC1-08	2,189,077	437,815,400	417,281,800	0.03, 0.03	97.62, 96.23	92.29, 90.61	35.31, 35.75
BC1-09	2,344,602	468,920,400	449,631,200	0.03, 0.03	97.63, 96.34	92.33, 90.83	35.25, 35.64
BC1-10	4,135,127	827,025,400	790,799,200	0.03, 0.03	98.01, 96.30	93.34, 90.93	35.15, 35.72
BC1-11	4,721,200	944,240,000	890,263,400	0.03, 0.03	97.67, 96.52	92.45, 91.25	35.29, 35.66
BC1-12	2,462,085	492,417,000	468,693,600	0.03, 0.03	98.06, 96.03	93.51, 90.80	35.96, 36.20

BC1-13	3,595,973	719,194,600	689,947,000	0.03, 0.03	97.71, 96.43	92.54, 91.07	35.27, 35.71
BC1-14	3,124,951	624,990,200	593,231,600	0.03, 0.03	97.66, 96.29	92.41, 90.79	35.18, 35.53
BC1-15	3,239,815	647,963,000	609,687,800	0.03, 0.03	97.76, 96.57	92.70, 91.40	35.30, 35.55
BC1-16	4,683,450	936,690,000	891,612,800	0.03, 0.03	97.68, 96.37	92.42, 90.91	35.28, 35.67
BC1-17	2,581,952	516,390,400	483,250,600	0.03, 0.03	98.08, 96.15	93.61, 91.14	35.98, 36.06
BC1-18	2,475,535	495,107,000	474,183,800	0.03, 0.03	97.71, 96.33	92.52, 90.83	35.22, 35.60
BC1-19	3,302,630	660,526,000	628,866,800	0.03, 0.03	97.73, 96.20	92.53, 90.55	35.21, 35.60
BC1-20	2,946,991	589,398,200	564,049,000	0.03, 0.03	97.73, 96.33	92.54, 90.82	35.19, 35.57
BC1-21	1,950,248	390,049,600	373,455,400	0.03, 0.03	97.57, 96.13	92.13, 90.40	35.20, 35.58
BC1-22	2,172,696	434,539,200	403,072,600	0.03, 0.04	97.88, 95.52	92.83, 89.01	36.26, 36.15
BC1-23	3,192,618	638,523,600	599,088,200	0.03, 0.03	98.10, 96.47	93.56, 91.32	35.23, 35.48
BC1-24	1,970,093	394,018,600	374,822,000	0.03, 0.03	98.06, 96.01	93.53, 90.77	35.93, 36.12
BC1-25	3,319,169	663,833,800	629,454,400	0.03, 0.03	97.97, 96.22	93.23, 90.77	35.15, 35.54
BC1-26	3,488,015	697,603,000	664,074,000	0.03, 0.03	97.95, 96.38	93.18, 91.06	35.22, 35.62
BC1-27	3,173,526	634,705,200	589,454,200	0.03, 0.03	98.06, 96.23	93.53, 91.22	35.99, 36.14
BC1-28	2,669,551	533,910,200	506,790,000	0.03, 0.03	98.07, 96.09	93.53, 90.93	35.97, 36.09
BC1-29	2,404,942	480,988,400	454,385,000	0.03, 0.04	97.83, 95.28	92.64, 88.53	36.33, 36.11
BC1-30	1,861,207	372,241,400	354,640,800	0.03, 0.03	98.05, 95.95	93.46, 90.62	35.92, 36.16
BC1-31	2,847,177	569,435,400	533,344,000	0.03, 0.04	97.79, 95.67	92.56, 89.27	36.44, 36.13
BC1-32	2,431,865	486,373,000	460,549,400	0.03, 0.04	97.87, 95.55	92.76, 89.06	36.28, 36.08
BC1-33	3,930,066	786,013,200	735,700,400	0.02, 0.03	98.59, 96.95	94.77, 92.10	35.55, 35.84
BC1-34	3,266,601	653,320,200	617,745,400	0.03, 0.04	97.84, 95.25	92.63, 88.48	36.32, 36.07
BC1-35	2,825,310	565,062,000	519,599,800	0.03, 0.04	97.74, 95.61	92.42, 89.14	36.52, 36.10
BC1-36	2,068,260	413,652,000	393,306,800	0.03, 0.03	97.96, 96.00	93.35, 90.76	35.92, 36.13
BC1-37	4,213,810	842,762,000	798,597,600	0.03, 0.04	97.84, 95.32	92.62, 88.58	36.31, 36.09
BC1-38	2,554,699	510,939,800	483,139,600	0.03, 0.04	97.87, 95.30	92.73, 88.57	36.21, 36.02
BC1-39	3,238,514	647,702,800	611,013,000	0.03, 0.04	97.83, 95.41	92.63, 88.78	36.31, 36.11
BC1-40	4,981,481	996,296,200	929,391,200	0.02, 0.03	98.56, 97.07	94.71, 92.38	35.47, 35.75
BC1-41	2,281,094	456,218,800	430,134,800	0.03, 0.04	97.84, 95.37	92.66, 88.71	36.61, 36.38
BC1-42	3,308,211	661,642,200	627,585,200	0.03, 0.04	97.87, 95.47	92.75, 88.90	36.32, 36.05
BC1-43	3,701,277	740,255,400	702,500,000	0.03, 0.04	97.87, 95.55	92.76, 89.05	36.51, 36.09
BC1-44	1,686,991	337,398,200	319,094,000	0.03, 0.03	98.05, 96.08	93.50, 90.92	35.89, 36.16
BC1-45	2,923,021	584,604,200	539,817,400	0.03, 0.03	98.09, 96.34	93.64, 91.47	36.36, 36.42
BC1-46	2,741,787	548,357,400	518,179,000	0.02, 0.03	98.46, 96.76	94.39, 91.67	35.47, 35.74
BC1-47	3,659,906	731,981,200	699,942,000	0.03, 0.03	98.03, 95.98	93.45, 90.69	35.93, 36.13
BC1-48	5,150,703	1,030,140,600	981,018,800	0.03, 0.03	97.99, 96.34	93.28, 90.99	35.19, 35.67
BC1-49	6,198,718	1,239,743,600	1,189,449,800	0.03, 0.03	97.98, 96.32	93.26, 90.95	35.14, 35.61

BC1-50	1,456,518	291,303,600	275,627,000	0.03, 0.04	97.83, 95.39	92.63, 88.72	36.40, 36.15
BC1-51	5,230,696	1,046,139,200	960,263,800	0.02, 0.03	98.53, 97.17	94.62, 92.56	37.27, 37.34
BC1-52	1,977,722	395,544,400	376,768,600	0.03, 0.03	97.98, 96.19	93.24, 90.71	35.12, 35.57
BC1-53	2,182,526	436,505,200	411,280,400	0.03, 0.03	98.07, 96.03	93.57, 90.84	35.86, 36.05
BC1-54	2,559,490	511,898,000	484,473,400	0.03, 0.03	97.99, 96.41	93.31, 91.14	35.20, 35.65
BC1-55	1,648,414	329,682,800	312,721,200	0.03, 0.03	98.09, 96.12	93.61, 91.00	35.91, 36.08
BC1-56	4,821,349	964,269,800	912,911,200	0.02, 0.03	98.57, 96.99	94.73, 92.20	35.40, 35.75
BC1-57	3,154,160	630,832,000	589,835,200	0.03, 0.03	98.06, 96.67	93.53, 91.75	35.32, 35.62
BC1-58	2,619,113	523,822,600	498,863,800	0.03, 0.03	98.02, 96.45	93.39, 91.22	35.23, 35.64
BC1-59	1,968,901	393,780,200	375,619,400	0.03, 0.03	97.94, 96.19	93.10, 90.68	35.14, 35.62
BC1-60	2,297,267	459,453,400	434,883,400	0.03, 0.03	98.01, 96.39	93.33, 91.13	35.12, 35.53
BC1-61	3,318,737	663,747,400	634,808,600	0.03, 0.03	97.96, 96.12	93.16, 90.55	35.17, 35.67
BC1-62	3,691,185	738,237,000	703,473,200	0.03, 0.03	98.04, 95.98	93.43, 90.67	36.00, 36.21
BC1-63	2,986,348	597,269,600	551,041,800	0.03, 0.04	97.66, 95.72	92.42, 89.62	35.18, 35.46
BC1-64	4,139,319	827,863,800	792,049,000	0.03, 0.03	97.96, 96.20	93.18, 90.73	35.21, 35.72
BC1-65	1,885,225	377,045,000	357,928,200	0.03, 0.03	97.96, 96.33	93.22, 90.98	35.46, 35.92
BC1-66	2,461,267	492,253,400	448,516,200	0.03, 0.04	97.68, 95.69	92.46, 89.58	35.23, 35.50
BC1-67	3,034,431	606,886,200	542,127,200	0.03, 0.04	97.61, 95.58	92.33, 89.36	35.35, 35.59
BC1-68	4,826,169	965,233,800	924,044,200	0.03, 0.03	97.99, 96.18	93.28, 90.69	35.26, 35.67
BC1-69	2,704,923	540,984,600	477,327,600	0.04, 0.05	96.67, 94.57	90.11, 86.86	40.26, 40.07
BC1-70	2,839,618	567,923,600	539,386,800	0.03, 0.03	98.06, 95.99	93.51, 90.72	35.99, 36.19
BC1-71	2,540,514	508,102,800	480,115,400	0.03, 0.03	98.08, 96.00	93.56, 90.76	35.83, 36.09
BC1-72	2,664,270	532,854,000	486,096,400	0.03, 0.04	97.90, 95.75	92.98, 89.57	35.37, 35.57
BC1-73	2,498,274	499,654,800	452,270,600	0.03, 0.04	97.83, 95.66	92.81, 89.41	35.39, 35.57
BC1-74	2,822,794	564,558,800	509,343,400	0.03, 0.03	97.85, 95.80	92.90, 89.69	35.41, 35.53
BC1-75	2,363,552	472,710,400	434,048,200	0.03, 0.03	97.94, 95.99	93.09, 90.03	35.50, 35.63
BC1-76	3,137,934	627,586,800	597,509,000	0.02, 0.03	98.53, 96.66	94.55, 91.46	35.53, 35.85
BC1-77	2,112,068	422,413,600	400,937,200	0.03, 0.03	97.93, 96.09	93.11, 90.50	35.17, 35.63
BC1-78	2,641,358	528,271,600	488,658,800	0.03, 0.04	97.52, 95.85	92.03, 89.74	35.25, 35.56
BC1-79	2,350,012	470,002,400	425,754,600	0.03, 0.03	97.90, 95.93	92.97, 89.90	35.24, 35.44
BC1-80	2,900,656	580,131,200	554,021,000	0.02, 0.03	98.56, 96.67	94.65, 91.46	35.49, 35.90
BC1-81	2,483,855	496,771,000	469,363,600	0.03, 0.03	98.01, 95.96	93.42, 90.70	35.98, 36.21
BC1-82	2,899,078	579,815,600	529,730,000	0.03, 0.04	97.81, 95.70	92.77, 89.42	35.44, 35.60
BC1-83	3,309,100	661,820,000	628,552,000	0.02, 0.03	98.56, 96.86	94.67, 91.89	35.29, 35.76
BC1-84	2,747,751	549,550,200	506,588,400	0.03, 0.04	97.45, 95.71	91.86, 89.50	35.26, 35.52
BC1-85	2,339,004	467,800,800	442,924,800	0.02, 0.03	98.56, 96.89	94.71, 92.00	35.26, 35.56
BC1-86	1,874,873	374,974,600	349,341,000	0.02, 0.03	98.55, 96.82	94.66, 91.87	35.38, 35.68

BC1-87	2,727,560	545,512,000	480,951,000	0.03, 0.04	97.43, 95.47	91.82, 89.09	35.31, 35.58
BC1-88	3,613,318	722,663,600	663,979,200	0.03, 0.03	97.88, 95.99	92.98, 90.05	35.56, 35.61
BC1-89	2,939,256	587,851,200	527,690,000	0.03, 0.03	97.50, 95.81	91.98, 89.75	35.26, 35.47
BC1-90	3,034,116	606,823,200	579,153,000	0.03, 0.03	97.66, 96.35	92.43, 90.89	35.16, 35.54
BC1-91	4,413,807	882,761,400	823,245,600	0.02, 0.03	98.55, 96.95	94.63, 92.09	35.71, 35.81
BC1-92	3,237,889	647,577,800	621,754,600	0.03, 0.03	97.68, 96.21	92.43, 90.58	35.25, 35.67
BC1-93	2,463,576	492,715,200	469,785,400	0.03, 0.03	98.00, 95.92	93.32, 90.54	35.93, 36.17
BC1-94	1,191,600	238,320,000	223,133,000	0.03, 0.04	97.86, 95.43	92.77, 88.83	36.04, 35.94
BC1-95	1,758,280	351,656,000	331,379,800	0.03, 0.04	97.84, 95.24	92.65, 88.47	36.21, 36.06
BC1-96	1,597,867	319,573,400	301,277,800	0.03, 0.04	97.87, 95.40	92.76, 88.80	36.18, 36.04
BC1-97	996,907	199,381,400	187,044,600	0.03, 0.04	97.85, 95.49	92.72, 88.95	35.99, 35.82
BC1-98	1,720,482	344,096,400	324,761,600	0.03, 0.04	97.80, 95.43	92.60, 88.81	36.34, 36.21
BC1-99	1,317,766	263,553,200	246,952,200	0.03, 0.04	97.78, 95.45	92.54, 88.86	36.19, 36.07
BC1-100	1,562,683	312,536,600	293,527,800	0.03, 0.04	97.77, 95.22	92.48, 88.40	36.27, 36.13

Supplementary Table S2 Clean reads and information mapped to the silkworm reference genome.

Samples	Clean read pairs	Rmduped read pairs	Read pairs with *EcoR* I recognition site
BC1-01	2,750,254	2,253,984	1,756,259
BC1-02	3,535,175	3,142,940	3,048,095
BC1-03	4,372,945	3,604,111	3,436,064
BC1-04	4,354,467	3,614,138	3,435,684
BC1-05	2,982,917	2,670,419	2,600,061
BC1-06	2,159,966	1,826,624	1,670,383
BC1-07	2,793,258	2,527,593	2,452,116
BC1-08	2,086,409	1,750,856	1,648,481
BC1-09	2,248,156	1,901,017	1,831,631
BC1-10	3,953,996	3,236,053	3,095,367
BC1-11	4,451,317	3,666,882	3,374,176
BC1-12	2,343,468	2,118,902	2,031,669
BC1-13	3,449,735	2,892,134	2,769,883
BC1-14	2,966,158	2,480,678	2,337,614
BC1-15	3,048,439	2,573,614	2,336,017
BC1-16	4,458,064	3,680,139	3,477,094
BC1-17	2,416,253	2,187,258	2,055,459
BC1-18	2,370,919	2,001,754	1,911,965
BC1-19	3,144,334	2,625,140	2,470,844

BC1-20	2,820,245	2,355,718	2,224,899
BC1-21	1,867,277	1,579,636	1,499,325
BC1-22	2,015,363	1,915,608	1,725,159
BC1-23	2,995,441	2,405,817	2,173,774
BC1-24	1,874,110	1,704,664	1,634,822
BC1-25	3,147,272	2,581,958	2,434,617
BC1-26	3,320,370	2,735,017	2,584,636
BC1-27	2,947,271	2,658,707	2,398,572
BC1-28	2,533,950	2,296,831	2,223,443
BC1-29	2,271,925	2,165,587	2,003,192
BC1-30	1,773,204	1,617,871	1,558,686
BC1-31	2,666,720	2,552,156	2,413,818
BC1-32	2,302,747	2,193,029	1,988,256
BC1-33	3,678,502	3,087,709	2,746,471
BC1-34	3,088,727	2,947,380	2,844,396
BC1-35	2,597,999	2,499,517	2,262,086
BC1-36	1,966,534	1,783,223	1,707,882
BC1-37	3,992,988	3,780,839	3,620,002
BC1-38	2,415,698	2,295,880	2,158,098
BC1-39	3,055,065	2,909,105	2,791,674
BC1-40	4,646,956	3,886,997	3,377,862
BC1-41	2,150,674	2,053,229	1,874,913
BC1-42	3,137,926	2,991,230	2,910,466
BC1-43	3,512,500	3,316,277	3,121,469
BC1-44	1,595,470	1,455,698	1,377,780
BC1-45	2,699,087	2,435,769	2,252,135
BC1-46	2,590,895	2,202,824	2,044,786
BC1-47	3,499,710	3,158,887	3,076,829
BC1-48	4,905,094	3,945,982	3,779,891
BC1-49	5,947,249	4,743,836	4,622,071
BC1-50	1,378,135	1,331,165	1,279,284
BC1-51	4,801,319	3,939,907	3,224,029
BC1-52	1,883,843	1,583,530	1,522,119
BC1-53	2,056,402	1,868,408	1,777,120
BC1-54	2,422,367	1,998,356	1,846,610
BC1-55	1,563,606	1,429,858	1,365,399
BC1-56	4,564,556	3,766,313	3,532,643

BC1-57	2,949,176	2,433,105	2,136,350
BC1-58	2,494,319	2,067,635	1,932,893
BC1-59	1,878,097	1,563,555	1,488,978
BC1-60	2,174,417	1,810,510	1,683,894
BC1-61	3,174,043	2,623,416	2,535,561
BC1-62	3,517,366	3,137,330	3,024,328
BC1-63	2,755,209	2,422,195	2,238,251
BC1-64	3,960,245	3,256,687	3,161,817
BC1-65	1,789,641	1,488,028	1,378,969
BC1-66	2,242,581	1,982,444	1,814,655
BC1-67	2,710,636	2,412,144	2,107,253
BC1-68	4,620,221	3,762,223	3,680,671
BC1-69	2,386,638	2,268,977	1,943,926
BC1-70	2,696,934	2,433,137	2,334,215
BC1-71	2,400,577	2,172,181	2,053,400
BC1-72	2,430,482	2,191,865	1,979,403
BC1-73	2,261,353	2,056,888	1,806,147
BC1-74	2,546,717	2,319,662	2,078,559
BC1-75	2,170,241	1,954,852	1,756,512
BC1-76	2,987,545	2,528,916	2,413,213
BC1-77	2,004,686	1,662,240	1,584,347
BC1-78	2,443,294	2,150,501	1,947,666
BC1-79	2,128,773	1,927,355	1,704,673
BC1-80	2,770,105	2,351,461	2,283,471
BC1-81	2,346,818	2,120,658	1,988,021
BC1-82	2,648,650	2,404,094	2,181,371
BC1-83	3,142,760	2,616,723	2,482,026
BC1-84	2,532,942	2,241,952	2,047,065
BC1-85	2,214,624	1,893,257	1,756,219
BC1-86	1,746,705	1,495,466	1,349,149
BC1-87	2,404,755	2,174,885	1,900,289
BC1-88	3,319,896	3,001,413	2,659,417
BC1-89	2,638,450	2,354,452	2,047,420
BC1-90	2,895,765	2,418,460	2,285,561
BC1-91	4,116,228	3,461,170	3,027,746
BC1-92	3,108,773	2,605,049	2,502,864
BC1-93	2,348,927	2,122,463	2,044,779

BC1-94	1,115,665	1,060,846	991,555
BC1-95	1,656,899	1,585,188	1,467,669
BC1-96	1,506,389	1,432,451	1,208,388
BC1-97	935,223	883,350	720,477
BC1-98	1,623,808	1,540,617	1,349,125
BC1-99	1,234,761	1,173,098	989,075
BC1-100	1,467,639	1,403,101	1,276,567

Supplementary Table S3 SNP numbers in the BC_1 mapping population.

Samples	Total SNP	Homo SNP	Hetero SNP	Homo rate (%)
BC1-1	102705	21509	81196	20.94
BC1-2	691490	406188	285302	58.74
BC1-3	791851	439048	352803	55.45
BC1-4	820263	432938	387325	52.78
BC1-5	592821	346019	246802	58.37
BC1-6	331553	161535	170018	48.72
BC1-7	587000	289114	297886	49.25
BC1-8	325778	174201	151577	53.47
BC1-9	436447	171490	264957	39.29
BC1-10	697847	396991	300856	56.89
BC1-11	797857	416412	381445	52.19
BC1-12	465236	228507	236729	49.12
BC1-13	693805	302579	391226	43.61
BC1-14	528046	286194	241852	54.20
BC1-15	544401	247445	296956	45.45
BC1-16	823597	441034	382563	53.55
BC1-17	447281	243566	203715	54.45
BC1-18	434954	203741	231213	46.84
BC1-19	605053	287646	317407	47.54
BC1-20	528773	244160	284613	46.17
BC1-21	321450	137051	184399	42.64
BC1-22	357512	163426	194086	45.71
BC1-23	394077	191947	202130	48.71
BC1-24	356489	164011	192478	46.01
BC1-25	508790	295503	213287	58.08
BC1-26	583232	299975	283257	51.43
BC1-27	552667	265768	286899	48.09
BC1-28	512226	262439	249787	51.24
BC1-29	447721	210575	237146	47.03
BC1-30	349103	140128	208975	40.14
BC1-31	581917	257534	324383	44.26
BC1-32	439627	198376	241251	45.12

BC1-33	626604	308180	318424	49.18
BC1-34	673024	330115	342909	49.05
BC1-35	501219	248448	252771	49.57
BC1-36	363748	178941	184807	49.19
BC1-37	911283	408873	502410	44.87
BC1-38	486432	235120	251312	48.34
BC1-39	640766	332310	308456	51.86
BC1-40	751492	344085	407407	45.79
BC1-41	457485	168696	288789	36.87
BC1-42	682470	349799	332671	51.25
BC1-43	698438	382506	315932	54.77
BC1-44	272751	119605	153146	43.85
BC1-45	493179	235633	257546	47.78
BC1-46	433680	216365	217315	49.89
BC1-47	807987	321198	486789	39.75
BC1-48	894292	454978	439314	50.88
BC1-49	1089693	594336	495357	54.54
BC1-50	255852	105045	150807	41.06
BC1-51	480835	215617	265218	44.84
BC1-52	336371	133871	202500	39.80
BC1-53	400397	181670	218727	45.37
BC1-54	394035	195361	198674	49.58
BC1-55	270567	121958	148609	45.07
BC1-56	811326	410653	400673	50.62
BC1-57	492907	208563	284344	42.31
BC1-58	434355	198170	236185	45.62
BC1-59	338507	117412	221095	34.69
BC1-60	373851	149523	224328	40.00
BC1-61	581284	291513	289771	50.15
BC1-62	691276	401003	290273	58.01
BC1-63	528670	245984	282686	46.53
BC1-64	744414	381080	363334	51.19
BC1-65	275682	115045	160637	41.73
BC1-66	408050	192681	215369	47.22
BC1-67	509492	224223	285269	44.01
BC1-68	840624	495402	345222	58.93
BC1-69	211504	77529	133975	36.66
BC1-70	526912	277120	249792	52.59
BC1-71	472125	210282	261843	44.54
BC1-72	485986	193605	292381	39.84
BC1-73	419804	172418	247386	41.07
BC1-74	492293	210337	281956	42.73
BC1-75	363204	189789	173415	52.25

BC1-76	515999	297088	218911	57.58
BC1-77	306404	169483	136921	55.31
BC1-78	449115	201498	247617	44.87
BC1-79	389110	146439	242671	37.63
BC1-80	512930	241982	270948	47.18
BC1-81	464538	203811	260727	43.87
BC1-82	521914	232086	289828	44.47
BC1-83	551350	271175	280175	49.18
BC1-84	469099	231274	237825	49.30
BC1-85	367572	171103	196469	46.55
BC1-86	269472	97892	171580	36.33
BC1-87	448278	196507	251771	43.84
BC1-88	613432	301640	311792	49.17
BC1-89	510330	199690	310640	39.13
BC1-90	547032	255421	291611	46.69
BC1-91	713969	327191	386778	45.83
BC1-92	570083	304370	265713	53.39
BC1-93	433382	242954	190428	56.06
BC1-94	179869	52985	126884	29.46
BC1-95	323221	117045	206176	36.21
BC1-96	236340	64079	172261	27.11
BC1-97	80472	11788	68684	14.65
BC1-98	256409	102016	154393	39.79
BC1-99	143167	44350	98817	30.98
BC1-100	243271	98495	144776	40.49

Supplementary Table S4 Summary of SNP-based linkage maps.

**Chromosome/ linkage group**	**Number of markers**	**Recombination length (cM)**	**Average length (cM)**
1	92	148.12	1.61
2	283	177.66	0.63
3	388	194.49	0.50
4	381	146.47	0.38
5	299	141.97	0.47
6	411	202.01	0.49
7	343	192.22	0.56
8	343	158.30	0.46
9	393	127.77	0.33
10	456	191.88	0.42
11	387	180.41	0.47
12	428	153.89	0.36
13	381	159.44	0.42
14	323	240.88	0.75

15	554	254.83	0.46
16	146	119.55	0.82
17	498	213.04	0.43
18	400	194.48	0.49
19	255	230.75	0.90
20	250	119.84	0.48
21	362	157.46	0.43
22	310	112.31	0.36
23	470	188.45	0.40
24	378	192.98	0.51
25	361	114.39	0.32
26	259	123.86	0.48
27	239	143.53	0.60
28	242	183.99	0.76
Total	9632	4764.96	0.49

Supplementary Table S5 Putative genes within QTL regions and expression profiles. The gene_IDs were retrieved from Silkbase (http://silkbase.ab.a.u-tokyo.ac.jp/) and SilkDB v2.0 (http://silkworm.genomics.org.cn/). The genes exhibiting a signal of artificial selection are shown in red. Functional annotation was performed via BLAST searches against the nr database (https://www.ncbi.nlm.nih.gov/) and PFAM (http://pfam.xfam.org/) with a threshold *E* value of ≤1e-5. In the developmental transcriptome of fifth-instar larvae between the domestic and wild silkworms, the expression levels of DEGs are also shown in red in the domestic silkworm. The transcription levels of all the putative genes in the various tissues on day 3 of the fifth-instar larave were retrieved from NCBI Sequence Read Archive database (Accession number PRJNA284192). Br, brain; ASG, anterior silk gland; AMSG, anterior section of middle silk gland; MMSG, middle section of middle silk gland; PMSG, posterior section of middle silk gland; PSG, posterior silk gland.

Supplementary Table S6 Candidate genes within regions of selective sweeps co-localizing with QTLs. The signals of a selective sweep were identified based on thresholds of F_{ST} >0.4514 and θ_π ratio <0.1835. Gene shown in red was also identified as candidate with signal of selective sweep in a previous study[1].

Gene_ID in SilkBase	Gene_ID in SilkDB v2.0	QTL	Chr.	Scaffold	Start	End
KWMTBOMO04947	BGIBMGA007997	qtl1-1	9	nscaf2889	2170421	2171420
KWMTBOMO04927	BGIBMGA008005	qtl1-1	9	nscaf2889	1770361	1770860
KWMTBOMO04918	BGIBMGA008009	qtl1-1	9	nscaf2889	1437434	1437850
KWMTBOMO04917	BGIBMGA008010	qtl1-1	9	nscaf2889	1423351	1423850
KWMTBOMO04914	BGIBMGA008011	qtl1-1	9	nscaf2889	1387851	1388850
KWMTBOMO04915	BGIBMGA008073	qtl1-1	9	nscaf2889	1392851	1393350

KWMTBOMO04915	BGIBMGA008073	qtl1-1	9	nscaf2889	1394351	1394850
KWMTBOMO15675	BGIBMGA002295	qtl1-3	26	nscaf2330	509511	510010
KWMTBOMO15678	BGIBMGA002280	qtl1-3	26	nscaf2330	714061	714560
KWMTBOMO15678	BGIBMGA002280	qtl1-3	26	nscaf2330	714561	715060
KWMTBOMO00426	BGIBMGA000486	qtl2-1	1	nscaf1690	7360451	7360950
KWMTBOMO00426	BGIBMGA000486	qtl2-1	1	nscaf1690	7360951	7361450
KWMTBOMO00426	BGIBMGA000486	qtl2-1	1	nscaf1690	7361451	7361950
KWMTBOMO00426	BGIBMGA000486	qtl2-1	1	nscaf1690	7361951	7362450
KWMTBOMO00380	BGIBMGA000511	qtl2-1	1	nscaf1690	6146331	6146830
KWMTBOMO00440	BGIBMGA000748	qtl2-1/qtl4-1	1	nscaf1690	7831691	7832190
KWMTBOMO13522	BGIBMGA001310	qtl2-3	23	nscaf1962	250571	251070
KWMTBOMO07656	BGIBMGA000854	qtl3-1	13	nscaf1898	14347381	14347880
KWMTBOMO12920	BGIBMGA000225	qtl4-3	22	nscaf1681	7359951	7360450

Supplementary Table S7 qPCR primer sequences and annealing temperatures. *RpL3* sequence was retrieved from GenBank under accession number NM_001043661.

Accession number/ gene name	Forward and reverse primers (5'-3')	Length of amplificon (bp)	Anneal temperature (°C)
KWMTBOMO12906	CGGAGGCTACAGAAGCAGATAAT<br>TTTAGTAACCAAAAATACAGCATCG	191	53
KWMTBOMO04917	GCATATCTTCAGGCACAACG<br>CATCAACAAAAGTTAAAGCTCCA	176	52
RpL3	CGGTGTTGTTGGATACATTGAG<br>GCTCATCCTGCCATTTCTTACT	161	55

Supplementary Table S8 miRNAs in QTL regions and potential targets encoding silk fibroin proteins. The miRNAs were retrieved from a previous study, which were expressed in the fifth-instar posterior silk gland of the domestic silkworm[2].

miRNA	Target gene	miRNA	Target gene
**qtl1-2**		bmo-miR-P939-5p	
bmo-miR-P499-5p		bmo-miR-P944-5p	
bmo-miR-P1017-5p		**qtl2-2**	
bmo-miR-P915-5p		bmo-miR-P886-5p	
**qtl1-3**		bmo-miR-P325-3p	
bmo-miR-P1487-3p		bmo-miR-P294-3p	
bmo-miR-P1298-3p		**qtl2-3**	
**qtl2-1**		bmo-miR-P228-3p	
bmo-miR-P009-3p	P25	bmo-miR-P1466-3p	

bmo-miR-P009-5p		**qtl2-4**	
bmo-miR-P020-3p		bmo-miR-P1192-5p	
bmo-miR-P020-5p		**qtl3-1**	
bmo-miR-P054-3p		bmo-miR-P723-3p	
bmo-miR-P054-5p		**qtl4-1**	
bmo-miR-P062-3p		bmo-miR-P062-3p	
bmo-miR-P100-3p		bmo-miR-P1416-3p	P25
bmo-miR-P1147-5p		bmo-miR-P786-3p	
bmo-miR-P1230-5p		bmo-miR-P939-5p	
bmo-miR-P1306-3p		bmo-miR-P1147-5p	
bmo-miR-P1326-3p		bmo-miR-P330-3p	
bmo-miR-P1416-3p	P25	bmo-miR-P623-5p	
bmo-miR-P1453-3p		bmo-miR-P1230-5p	
bmo-miR-P1481-3p		bmo-miR-P009-5p	
bmo-miR-P330-3p		bmo-miR-P009-3p	P25
bmo-miR-P343-3p		bmo-miR-P1481-3p	
bmo-miR-P350-5p		**qtl4-2**	
bmo-miR-P490-5p		bmo-miR-P1415-3p	
bmo-miR-P581-5p	P25	bmo-miR-P953-5p	
bmo-miR-P623-5p		**qtl4-3**	
bmo-miR-P786-3p		bmo-miR-P1415-3p	
bmo-miR-P858-5p		bmo-miR-P953-5p	

References

1. Xia, Q. *et al.* Complete resequencing of 40 genomes reveals domestication events and genes in silkworm (*Bombyx*). *Science* 326, 433-436 (2009).
2. Li, J. *et al.* MicroRNA expression profiling of the fifth-instar posterior silk gland of *Bombyx mori. BMC Genomics* 15, 410; 10.1186/1471-2164-15-410 (2014).
